# Can Support Groups Improve Treatment Adherence and Reduce Sexual Risk Behavior among Young People Living with HIV? Results from a Cohort Study in South Africa

**DOI:** 10.3390/tropicalmed9070162

**Published:** 2024-07-19

**Authors:** Tonya R. Thurman, Brian Luckett, Babalwa Zani, Johanna Nice, Tory M. Taylor

**Affiliations:** 1 Tulane International, Cape Town 7806, South Africa; 2 Highly Vulnerable Children Research Center, Department of International Health and Sustainable Development, School of Public Health and Tropical Medicine, Tulane University, New Orleans, LA 70112, USA; 3 Carolina Population Center, University of North Carolina at Chapel Hill, Chapel Hill, NC 27516, USA; torymt@unc.edu

**Keywords:** HIV, adolescents, youth, support group, adherence, sexual health, risk behavior

## Abstract

Adolescents and young adults living with HIV (AYLHIV) in sub-Saharan Africa experience poorer HIV outcomes than adults, including lower ART adherence and virologic suppression. They also have high rates of unprotected sex, increasing the risk of adverse sexual health outcomes and onward transmission. This one-arm, pre–post study investigates a structured 14-session support group aiming to boost treatment adherence and sexual protective behavior for AYLHIV in South Africa. Logistic and Poisson regression analyses were performed on self-reported pre- and post-intervention survey data collected approximately 7.5 months apart from a cohort of 548 AYLHIV. Participants were significantly more likely to report at least 95% adherence at follow-up and rate their health as “good;” they also demonstrated greater treatment knowledge and had fewer absences from school overall and due to illness. Among sexually active AYLHIV, contraception use at last sex increased significantly, while condom use did not. Effects were small, and greater programmatic emphasis on adherence and multifaceted service packages is likely necessary to promote viral suppression. Nonetheless, the intervention addresses an important gap in service provision for AYLHIV in South Africa. Findings denote the potential for incorporating care and treatment components into sexual and reproductive health interventions tailored for AYLHIV.

## 1. Introduction

South Africa has made significant progress towards the UNAIDS 95-95-95 targets: an estimated 90% of people aged 15 years or older living with HIV are aware of their status, 91% of those aware of their status are on antiretroviral treatment (ART), and 94% of those on ART are virally suppressed [1]. However, gaps in the care cascade among adolescents and young adults living with HIV (AYLHIV) in South Africa remain, with multiple studies reporting lower viral suppression in this group compared to older adults [2,3,4]. These results are consistent with other findings from sub-Saharan Africa (SSA) that suggest that adherence to ART, one of the strongest predictors of viral suppression [5], is lower among AYLHIV compared to adults and children [6,7,8]. One study among a cohort of adolescents from South Africa reported adherence rates as low as 36% [9].

Poor viral load suppression poses expanded risks as AYLHIV become sexually active. Two systematic reviews substantiate high rates of unprotected sex among AYLHIV in SSA, elevating their risk for unplanned pregnancy, sexually transmitted infections, and onward HIV transmission [10,11]. Research among AYLHIV in South Africa found lower condom use among those with poor treatment adherence, posing serious implications for both individual health and epidemic control [12]. Unprotected sex in the absence of viral suppression increases risk for HIV transmission between sexual partners, including HIV superinfection [13]. These findings denote a critical need among AYLHIV for sexual and reproductive health interventions alongside those supporting treatment adherence.

There is a dearth of operations research to address the programmatic needs of AYLHIV in SSA. Results from several systematic reviews highlight the scarcity of evaluated interventions from SSA targeting AYLHIV adherence [14,15,16]. Available findings suggest the value of tailoring interventions for AYLHIV specifically. For example, adherence clubs in South Africa have shown positive effects on viral suppression and retention in care for adults [17,18] but mixed effects among adolescents and young adults [19,20,21]. Further, while studies examining sexual and reproductive health interventions for young people in sub-Saharan Africa and other low- and middle-income contexts are plentiful [22,23], AYLHIV have unique needs related to status disclosure, stigma and discrimination, fear of violence, and other factors [24]. Few studies have evaluated sexual health programs among AYLHIV in SSA [10,25]. This cohort study aims to add to the evidence base by investigating the effects of a structured support group intervention on treatment-related outcomes and sexual behavior of AYLHIV in South Africa.

## 2. Materials and Methods

### 2.1. Intervention

Vhutshilo 3 is a curriculum-based prevention education peer support group for AYLHIV implemented by community-based organizations in South Africa with funding from the U.S. President’s Emergency Plan for AIDS Relief (PEPFAR) through its orphans and vulnerable children (OVC) programming [26]. Vhutshilo 3 aims to promote ART adherence and prevent sexual risk behaviors. It is a structured curriculum designed to be delivered by a trained facilitator to a closed group of 15 to 20 adolescents aged 14 years and older. It was adapted from earlier Vhutshilo interventions focused on HIV prevention with widescale implementation in South Africa since 2007. Results from evaluations of the original Vhutshilo model illustrated its potential to improve attitudes and knowledge pertaining to HIV transmission [27] and mitigate sexual risk behaviors when delivered in combination with an evidence-informed mental health intervention [28]. The Vhutshilo 3 intervention under study comprised 14 one-hour sessions, with each session typically delivered once every two weeks. Following a detailed curriculum, each session had a specific focal point, covering the sexual and reproductive health and psychosocial content from the original Vhutshilo models and additional sessions specific to AYLHIV. Session focal points included finding support, making healthy decisions, positive dating relationships, avoiding teen pregnancy through the use of condoms and contraception, coping with HIV and stigma, ART adherence, and onward transmission. Guided by a trained facilitator, participants engage in critical reflection on these topics through following a fictionalized narrative that includes complex, realistic adolescent characters. Further information about the Vhutshilo 3 model and its iterations (including reduction to 10 sessions, session titles, and an example session) is available on the developer’s website [29].

### 2.2. Study Design and Participants

This was a pragmatic, one-arm, pre- and post-intervention study conducted in urban communities in the KwaZulu-Natal and Gauteng provinces of South Africa: areas with the highest number and prevalence of adolescents living with HIV in the country [4]. Participants aged 14–24 and aware of their HIV status were recruited by community-based organizations serving AYLHIV to participate in Vhutshilo 3. Program recruitment was conducted separately from study recruitment, and beneficiaries who did not consent to the study remained eligible for participation in the intervention. Members of all support groups that commenced operation between June 2019 and March 2020 at each eligible site were invited to participate in the baseline survey.

### 2.3. Study Procedures

Data collection for this analysis began in June 2019 and ended in March 2020, when group sessions ceased due to COVID-19 precautions. Throughout this 10-month study period, participant enrolment was staggered with baseline surveys occurring as new groups were initiated and follow-up surveys scheduled accordingly at the last intervention session, generally about 7 months after the baseline survey. Sixty-two groups across 29 locations completed the intervention and both survey rounds (baseline and follow-up). A larger intervention cohort was planned but could not be completed due to the South African national lockdown; details on the full baseline sample and post-pandemic follow-up are reported elsewhere [30,31].

The Pharma Ethics Independent Research Ethics Committee in South Africa (study reference no: 181021579) and Social Behavioral Institutional Review Board at Tulane University (study reference no: 2018-1736) approved this study. Written voluntary informed consent was obtained from each study participant, and ethical approval included authorization for eligible children under age 18 to consent to their own participation, given that not all had disclosed their HIV status to their parent or caregiver.

Survey data collection was facilitated by an external research team during the first or second support group session. This arrangement was designed to ensure standardized administration and reduce participant burden. Each question was read aloud by survey staff while participants, seated apart so they could not see each other’s responses, marked individual copies of the questionnaire. While the written survey was in English, survey staff offered clarification upon request using a reference copy of the questionnaire locally translated into isiZulu and Setswana, common languages in the study areas. To ensure confidentiality and minimize social desirability bias, coded identifiers were used in place of names on the questionnaire, and each completed questionnaire was sealed in an individual envelope by the participant before returning it to survey staff. Participants received refreshments and a small monetary incentive (ZAR 20, approximately US$1.20) upon completing the survey. This study was a pragmatic trial under real-world conditions; as such, the study team did not influence the conditions of the intervention.

### 2.4. Measures

Participants’ age at the time of the survey was calculated using the date of birth recorded during registration with the program implementers; gender was also obtained from program registration records. All other measures were derived from survey responses. The survey covered measures of ART adherence, physical health, school attendance, knowledge pertaining to HIV treatment and care, as well as sexual behavior among participants who reported being sexually active.

Adherence was assessed by asking, “In the last month (30 days), how many days did you miss taking one of more of your ARV pills?” with space to write in the number or select “I’m not taking ARVs.” Local program staff suggested using the term ARV (antiretrovirals), indicating that it was commonly used by adolescents to describe antiretroviral therapy (ART). Being ART-adherent was defined as missing an ART dose for no more than one day in the previous month (95% adherence), consistent with other research [32,33]. This outcome included participants who were currently taking ART; those who never initiated ART or who reported not taking ART in the previous month were not included.

Participants also rated their health using a single measure, “How has your overall health been in the last month?” [34]. The response options were reduced from four to five to accommodate self-administration among adolescents and to avoid over-response on a middle, neutral, option.

School attendance was measured with two indicators of recent attendance and absences due to illness. Past week school attendance used the question, “During the last full school week, did you miss any days of school?” School missed due to illness used the question, “In the last month, have you missed any days of school because you were too sick to attend?”

HIV treatment knowledge was assessed with six survey items (responses combined into a single score). Three questions were adapted from earlier research among AYLHIV in South Africa [35] focused on ART use and asked about sharing pills, what to do if a pill is vomited, and if additional ART should be taken if a pill is missed. Two additional questions asked respondents for their interpretation of CD4 and viral load tests with respect to the immune system. The last question, drawn from the South Africa Demographic and Health Survey (DHS) [36], asked if a person could have HIV and not have AIDS.

Condom and other contraceptive use at last sex was collected among participants who reported having sex in the six months prior to each survey round to capture behavioral changes potentially associated with program engagement. Contraceptive use in this study included anything other than a condom, which was asked separately. Questions from the South Africa DHS [36] asked if they or their partner had used a condom or other form of contraception at last intercourse.

### 2.5. Statistical Methods

Frequencies, means, *t*-tests and chi-square tests were generated using SAS version 9.4. Regression models were generated in Stata IC version 14 using multilevel mixed-effects generalized estimation models with the MEGLM command. Dichotomous outcomes were modeled using the logit distribution and reported as odds ratios, while count data were modeled using the Poisson distribution and reported as incident rate ratios. Standard errors were corrected for repeated measures on the same individual. Intraclass correlation among intervention groups was tested and found to be non-significant and, therefore, not accounted for in the models. Records with missing data for the outcome were excluded from analyses.

All models included covariates for the age of the participant at baseline, their gender, the baseline value of the outcome being modeled, and a dummy variable for survey round with the follow-up survey round coded one and the baseline survey round coded zero. The models were also controlled for the number of days from completing the baseline questionnaire to completing the follow-up questionnaire to account for differences in program implementation schedules. The program effect estimation is the exponentiated coefficient of the survey round dummy variable. For the knowledge outcomes, only the scale scores were tested using Poisson regression, while differences in individual questions were compared using logistic regression.

Multiple potential confounding variables were tested, but none were found to alter the program impact measure by more than 10%. Additional, extensive moderation analyses were conducted, but none were found to be statistically significant.

Effect sizes were calculated using the Cohen’s d statistic for repeated measures as [37]. Cohen’s *d* statistics were classified as ‘no effect’ if the absolute value is less than 0.2 and ‘small effect’ if between 0.2 and 0.4.

## 3. Results

Cohort data came from 62 Vhutshilo 3 groups held at 29 locations. Among those 62 groups, 699 participants completed the baseline survey while 548 completed the follow-up survey. Those 548 participants who completed both surveys comprise the cohort sample.

Table 1 displays the characteristics and study outcomes of participants, and the subgroups of participants taking ART, attending school, and sexually active. Baseline sample characteristics are provided for all of the 62 Vhutshilo 3 group participants (*n* = 699) and the entire cohort sample of 548 to examine how well the cohort represents all group participants. For all characteristics presented in Table 1, the cohort sample was not statistically different than all baseline respondents in that group. Table 2 provides odds ratios and incident rate ratios for the program impact on the outcomes and their effect sizes for the cohort.

Almost 66% of cohort participants were female, and the mean age at baseline was 16.8 years (s.d. = 2.0 years); 89% were enrolled in school. The average time from baseline survey to follow-up survey was approximately 7.5 months (227.6 days, s.d. = 22.6 days).

At baseline, 87% of participants had initiated ART, and 73% reported having taken ART in the previous month, with 53% of participants categorized as at least 95% adherent to ART in the past month. The odds of being 95% adherent more than doubled at follow-up (OR = 2.21, 95% CI = 1.43–3.41). However, the effect size was small and only resulted in a 10% increase in participants reporting 95% optimal adherence.

Self-rated good health improved significantly after program participation with a 168% increase in the odds of reporting good or very good health (OR = 2.68, 95% CI = 1.95–3.70) and a 63% drop in the odds of reporting poor health (OR = 0.37, 95% CI = 0.20–0.71). School absences also showed significant improvement at follow-up with a reduction in the odds of missing school due to illness of 64% (OR = 0.36, 95% CI = 0.25–0.51) and reduced odds of missing school for any reason by 51% (OR = 0.49, 95% CI = 0.35–0.70).

The HIV treatment knowledge score showed a statistically significant 9% increase in the odds of answering an additional item correctly at follow-up (IRR = 1.09, 95% CI = 1.02–1.17). Analysis of individual items suggests that this improvement is primarily driven by changes in four items: knowing that someone can be HIV-positive but not have AIDS (47% to 51%: OR = 1.29, 95% CI = 1.96–1.73), knowing that a high viral load means a weak immune system (32% to 42%: OR = 1.88, 95% CI = 1.39–2.53), knowing that a high CD4 count means a strong immune system (33% to 41%: OR = 1.62, 95% CI = 1.21–2.17), and knowing not to take extra ART following a missed dose (75% to 80%: OR = 1.51, 95% CI = 1.05–2.17).

Among those study participants who had sex in the six months prior to the survey, there was a significant increase in contraceptive use (46% to 55%: OR = 2.33, 95% CI = 1.16–4.69) but not for condom use.

## 4. Discussion

This study found improvements in key outcomes among AYLHIV in South Africa participating in a structured group-based prevention education intervention. The most robust changes included better self-rated health and school attendance, important indicators of participants’ well-being and future prospects. Longitudinal research has illustrated the validity of self-rated health in reflecting adolescents’ physical and emotional wellness [38]; poor health on these dimensions contributes to stark educational deficits among adolescents living with HIV in South Africa [39]. Access to school has also been associated with lower prevalence of unprotected sex among AYLHIV in South Africa [40]. Better health and school attendance may be attributable to a combination of the psychosocial peer support received and improved treatment adherence. Participants had greater HIV treatment knowledge and were more likely to have optimal ART adherence. Effects on adherence were small, consistent with results from a recent meta-analysis showing small-to-moderate effects of psychosocial intervention studies on AYLHIV adherence to ART [41].

Significant care and treatment gaps remained. Almost thirty percent of cohort participants reported not being on ART, with no change following intervention engagement. While statistically significant improvements in 95% adherence were found among those on ART, one-third continued to have suboptimal adherence. Similarly, positive effects on knowledge pertaining to HIV treatment were small, and more than half of participants continued not to understand the basic influence of viral loads or CD4 counts on their immune system. Future interventions may benefit from increased emphasis on HIV care management and adherence to achieve greater targeted effects on clinically significant outcomes. Earlier research among this baseline sample suggested the value of screening tools for identifying those with greater adherence challenges, and discussion of these barriers could be integrated into the peer group discussion and used to identify those in need of more targeted support [31].

Contraception use also significantly increased among sexually active study participants, although condom use did not. Detection of increased sexual protective behavior is encouraging given the short time frame and small sample for this study, denoting the potential for sexual and reproductive health interventions tailored for AYLHIV. Indeed, earlier studies have shown improvements in condom use among AYLHIV in SSA participating in secondary HIV-prevention intervention studies [25]. Thorough examination of sexual risk behavior is limited by this study’s duration and small sample size, including an inability to examine likely differential gender effects. Moreover, combinations of interventions are likely to have the greatest effect on sexual risk-taking, as suggested in prior intervention research among AYLHIV [10] and earlier studies investigating the effects of the original Vhutshilo model among HIV-negative adolescents [28].

Several study limitations should be considered when interpreting results. Although self-reported rates of sexual behavior are higher than with traditional interviewer-initiated data collection [42] and multiple studies have found correlations between self-reported ART adherence and viral suppression [43], recall and social desirability bias are persistent limitations. At the same time, greater effects may have been apparent with a larger cohort and longer follow-up period, including annual viral load changes and sexual risk reduction. Loss to follow-up, at 21%, may have also introduced bias. While little difference was found on characteristics and outcomes under investigation between those who completed both survey rounds and those who did not, participants lost to follow-up may have unobserved differences, and this group includes both early dropouts and those who merely skipped the final session. Lack of linked attendance data limits understanding of this issue and prohibits dose–response analyses. However, data from this study likely reflect routine attendance levels broadly representative of the intended participants. Evidence from other studies of group-based interventions in South Africa also points to pervasive challenges restricting attendance and retention [44,45].

This cohort study occurred in real-world circumstances rather than the optimal conditions often applied in randomized controlled trials. Youth were recruited to participate in the survey from peer support groups organized and implemented by community-based organizations, extending the generalizability of findings beyond clinical samples [35]. Range in quality of implementation, variable attendance among participants, and other conditions outside of the control of researchers likely affected this pragmatic trial. While the absence of a control group limits the ability to rigorously attribute the results to the intervention under study, the effects of an intervention applied in routine practice exhibit greater external validity and hold unique value for identifying program gaps and possibilities. 

This study was initially designed to involve a larger cohort and longer follow-up period, inclusive of annual viral load results and examining community-based case management services available to participants following the group-based intervention. The COVID-19 pandemic impacted the extent of this work, abruptly interrupting the scale-up of the group-based intervention under study and participants’ access to these services. Effect sizes from the short-term evaluation of the support group were small, and greater emphasis on clinical outcomes and multifaceted service packages are likely necessary to promote viral suppression. 

## 5. Conclusions

Future studies should investigate combination service packages and include a broader array of service and clinic data with longer follow-up periods. Findings from this study add to the scarce interventional research targeting AYLHIV [46]. Results illustrate the potential of a group-based support intervention to address an important gap in service provision for AYLHIV in South Africa, uniquely providing both treatment support and prevention education with critical individual and public health implications.

## Figures and Tables

**Table 1:** Study participants’ characteristics and outcomes.

	Sample (n = 699)		Cohort (n = 548)			
	Baseline		Baseline		Follow-Up	
Outcome	n (Mean)	% (SD)	n (Mean)	% (SD)	n (Mean)	% (SD)
Age	(16.7)	(1.96)	(16.8)	(2.0)	(17.8)	(2.0)
Female	449	64.2	360	65.7	360	65.7
Days from baseline to follow-up survey	n/a				(227.6)	(22.6)
Taken ART in previous 30 days	499	71.4	400	73.0	394	71.9
Missed less than one ART dose in previous month (95% adherence)	246	49.3	201	52.8	235	62.7
Rated health as good or very good	382	54.6	297	54.4	376	69.1
Rated health as poor	54	7.7	44	8.1	25	4.6
Attending school	612	87.6	481	87.8	469	85.6
Missed any days of school last month due to illness	210	34.3	173	37.0	100	21.5
Missed any days of school in last week of school	234	38.2	180	37.4	126	26.9
HIV treatment knowledge score	(2.7)	(1.4)	(2.8)	(1.4)	(3.0)	(1.5)
Knows not to share ARTs	514	73.5	404	73.7	412	75.2
Knows to take another ART if vomited	113	16.2	93	17.0	81	14.8
Knows not to take extra ART if missed	521	74.5	413	75.4	437	79.7
Knows someone can be living with HIV but not have AIDS	321	45.9	255	46.5	278	50.7
Knows high VL means weak immune system	221	31.6	176	32.1	232	42.3
Knows high CD4 means strong immune system	224	32.1	180	32.9	225	41.1
Sex in past 6 months	176	25.2	143	26.1	128	23.4
Used a condom at last sex	133	75.6	105	74.5	98	76.0
Used contraception at last sex	77	43.8	66	46.2	70	54.7

**Table 2:** Regression results showing change over time among intervention cohort participants.

	Program Impact	95% CI			
Outcome	OR (IRR)	Lower	Upper	*p*-Value	Cohen’s d
Missed less than one ART dose in previous month (95% adherence)	2.21	1.43	3.41	0.000	0.153
Rated health as good or very good	2.68	1.95	3.70	0.000	0.227
Rated health as poor	0.37	0.20	0.71	0.003	−0.091
Missed any days of school last month due to illness	0.36	0.25	0.51	0.000	−0.159
Missed any days of school in last week of school	0.49	0.35	0.70	0.000	−0.111
HIV treatment knowledge score	(1.09)	1.02	1.17	0.011	0.152
Knows not to share ARTs	1.12	0.81	1.54	0.509	0.024
Knows to take another ART if vomited	0.78	0.53	1.16	0.228	−0.044
Knows not to take extra ART if missed	1.51	1.05	2.17	0.028	0.081
Knows someone can have HIV but not have AIDS	1.29	0.96	1.73	0.087	0.065
Knows high VL means weak immune system	1.88	1.39	2.53	0.000	0.164
Knows high CD4 means strong immune system	1.62	1.21	2.17	0.001	0.127
Used a condom at last sex	1.27	0.56	2.88	0.564	0.026
Used contraception at last sex	2.33	1.16	4.69	0.017	0.108

## Data Availability

The data presented in this study are available on reasonable request from the corresponding author. The data are not publicly available due to privacy or ethical restrictions.
